# Study on mechanism of Zhenwu Decoction in treatment of heart failure based on network pharmacology: A review

**DOI:** 10.1097/MD.0000000000036073

**Published:** 2023-11-17

**Authors:** Sai Yan, Qingchun Shi, Hongtao Ma, Qian Xu

**Affiliations:** a The second Affiliated Hospital of Heilongjiang University of Traditional Chinese Medicine, Harbin, China; b Baotou Mongolian Medicine Hospital of Traditional Chinese Medicine, Baotou, China.

**Keywords:** heart failure, network pharmacology, Zhenwu Decoction

## Abstract

To explore the mechanism of Zhenwu Decoction (ZWD) in the treatment of heart failure (HF) by network pharmacology analysis, so as to provide a basis for the innovation and application of drugs. The effective components and targets of 5 Chinese herbal medicines in ZWD were retrieved by TCM Pharmacology Database and Analysis Platform (TCMSP).Gene card, OMIM and TTD databases were used to obtain the disease targets of HF, and the intersection with the targets of ZWD was obtained. We used Cytoscape3.9.1 software to construct a drug-active component-disease-target interaction network for ZWD treatment of HF, and performed protein-protein interaction (PPI) network and topology analysis. Kyoto Encyclopedia of Genes and Genomes (KEGG) and Gene Ontology (GO) enrichment analyses were performed. Fifty-nine effective components and 229 targets of ZWD were screened. Among them, ZWD for HF has 27 active components and 38 common targets, and the core targets of PPI are IL-6, ATK1 and TNF. Pathway enrichment analysis included lipid and atherosclerotic and TNF signaling pathways. This study preliminarily clarified the main active components, targets and related pathways of ZWD in the treatment of HF, and laid a foundation for further study of the pharmacological effects of ZWD.

## 1. Introduction

Heart failure (HF) is a clinically prominent and common syndrome characterized by structural and/or functional abnormalities of the heart, resulting in increased intracardiac pressure and inadequate cardiac output. The common causes of HF are cardiac systolic dysfunction and relaxation. Zhang Dysfunction Heart failure can also be caused by pathological changes in the heart valves, pericardium, and abnormal heart rhythm.^[1]^ HF is a disease characterized by high morbidity and mortality, poor social capacity and quality of life, and high costs of treatment and prevention. HF affects more than 64 million people worldwide. One of the major issues in global public health today includes efforts to reduce the social and economic burden of HF.^[2]^ According to the statistics of China Research Association in 2019, the mortality rate of cardiovascular diseases in rural and urban areas accounted for 46.74% and 44.26% of all deaths. It is estimated that there are about 330 million patients with different degrees of cardiovascular disease in China, of which about 8.9 million are HF. At the same time, China is facing the dual pressure of population aging and the steady rise in the prevalence of metabolic risk factors. In the face of the increasing burden caused by cardiovascular diseases, new challenges have emerged in the prevention and treatment of cardiovascular diseases and the allocation of medical resources in China.^[3]^ According to the latest epidemiological data, there are about 12.1 million HF patients among people aged 25 and above in China, and there are about 3 million new HF patients every year, which has brought considerable burden to China health system.^[4]^

Currently, drugs such as angiotensin-converting enzyme inhibitors (ACEI), β blockers and aldosterone receptor antagonists are used to treat heart failure, which can effectively treat heart failure and delay the progression of heart failure, but caution in patients with severe renal dysfunction. Therefore, the new treatment regimen is to replace ACEI drugs with sakubarase, whose mechanism of action is to antagonize angiotensin II receptors and neuropilin and to effectively inhibit the activation of the neuroendocrine system, as recommended by many guidelines for clinical symptoms and long-term outcomes in patients with chronic heart failure.^[5,6]^

The study of HF in traditional Chinese medicine has a long history. Through medium and long-term clinical evidence, the disease position of HF is the deficiency of qi, blood and Yin and Yang, the loss of the function of viscera is the essence, blood stasis, phlegm, water drinking, qi stagnation, etc, and the blood obstruction is the standard. Its treatment is mainly to warm Yang and promote diuresis, so it is often treated with Zhenwu Decoction (ZWD). ZWD comes from Treatise on Febrile and Miscellaneous Diseases by Zhang Zhongjing, an outstanding physician in the Han Dynasty. The original text is “Taiyang disease, sweating, sweating, people still have fever, palpitation, dizziness, body movement, and desire to vibrate, Zhenwu Tang governs it.” Which consists of 5 medicines of tuckahoe, white paeony root, largehead Atractylodes rhizome, ginger and aconite. ZWD has the effects of warming and activating Yang qi and promoting urination, and is mainly used to treat edema caused by heart and kidney Yang deficiency. ZWD is especially suitable for the treatment of total heart failure, acute heart failure, heart failure with reduced ejection fraction, etc.^[7]^ ZWD has the effects of promoting urination, dilating blood vessels and strengthening heart, but its specific mechanism has not been clarified by special studies.

Traditional Chinese medicine (TCM) compound is a good resource for new drug development, and its clinical efficacy has been confirmed in thousands of years of clinical practice of traditional Chinese medicine, which is expected to bring benefits to patients with heart failure, but its multi-component, multi-target and multi-effect mechanism of action is a difficult problem in traditional Chinese medicine research. Network pharmacology integrates the emerging concepts and methods of systems biology, multi-directional pharmacology, computational biology and network analysis to explore the relationship between drugs and diseases from multiple levels, which has a certain degree of systematicness and integrity. Therefore, we used the TCMSP database to screen the active ingredients of ZWD and predict their targets. Under the guidance of network pharmacology, the interaction between ZWD active components and targets was analyzed, and the ZWD active component-target network and ZWD active component-target-HF target network were established. Finally, bioinformatics analysis was used to clarify the multi-target and multi-pathway mechanism of ZWD in the prevention and treatment of HF.

## 2. Materials and methods

### 2.1. Excavation and screening of active components and targets of ZWD

Through the application of TCMSP database (https://old.tcmsp-e.com/tcmsp.php), 5 kinds of traditional Chinese medicines, including aconite, tuckahoe, white Atractylodes rhizome, ginger and white peony root, were retrieved in turn. The screening conditions were set as (oral bioavailability, OB) OB ≥ 30% and (Drug Likeness, DL) ≥ 0.18 to obtain the corresponding active ingredients of the 5 Chinese herbal medicines, and the UniProt database (https://www.uniprot.org/) was used. UniProtKB search function, the database selection is limited to “Reviewed (Swiss-Prot),” the species is “Human,” and the target is queried to obtain the target corresponding to the active ingredient of ZWD.

### 2.2. Mining gene targets of HF

HF disease target are identified and identify through that Gene card database (https://ngdc.cncb.ac.cn/databasecommons/database/id/723), OMIM database (https://www.omim.org/) and https://ngdc.cncb.ac.cn/databasecommons/database/id/4948). The higher the Relevance score in the Gene cards database, the closer the relationship between the target and the disease, so the target with Relevance score ≥ 10 was taken as the target of HF disease.^[8]^ The key words “heart failure” or “HF” were used to search for the HF gene targets that had been confirmed and had related studies, and the duplicate and false positive genes were eliminated, which were the related gene targets of HF.

### 2.3. Construction of drug-active component-target interaction network for ZWD treatment of HF

The target of ZWD and the gene target of HF were imported into the UniProt database for standardization, and then the intersection target was screened as the potential target of ZWD for HF. The potential targets of ZWD were imported into the STRING: functional protein association networks database (https://cn.string-db.org/), and the Multiple proteins tool was used. Homo sapiens is defined as a species, the highest confidence is more than 0.4, protein interaction is obtained, the free protein is hidden, and the protein is obtained. Cytoscape software draws the protein-protein interaction network, analyzes the topology of the network, and visualizes the core target data to construct the PPI network diagram. Cytoscape3.9.1 software was used to construct the drug-active ingredient-disease-target interaction network of ZWD in the treatment of HF, and based on this network, the mechanism of action of the whole ZWD compound was explored.

### 2.4. Pathway enrichment analysis of GO and KEGG for ZWD treatment of HS targets

The DAVID database (https://david.ncifcrf.gov/) was used for enrichment analysis of targets for ZWD treatment of HF by Gene Ontology (GO) biological processes and metabolic pathways in Kyoto Encyclopedia of Genes and Genomes (KEGG). The molecular mechanism of ZWD acting on HF was elucidated from 2 aspects of gene function and pathway analysis.

### 2.5. Molecular docking

The core active ingredients screened from the “Drug-Active Ingredient-Disease-Target” network were selected for molecular docking with the core targets. The 3-dimensional structures of the potential targets and the screened targets to be verified are searched and obtained through the RCSB PDB database (http://www.rcsb.org/), The 3D structure of the ZWD core active compound was downloaded from the Pubchem database (https://pubchem.ncbi.nlm.nih.gov/), and AutoDockTools 1.5.7 software was used to remove water molecules and ligands from the above file, and at the same time, hydrogenation and electron addition were carried out. Carry out molecular docking on that core active ingredient and the core target.

## 3. Result

### 3.1. Prediction of active components and targets of ZWD

The TCMSP database was used to screen the active ingredients of ZWD, which met the screening statistics of OB value ≥ 30% and DL value ≥ 0.18, and 61 active ingredients were obtained. Among them, there are 21 kinds of effective components from Radix Aconiti Lateralis Preparata, 15 kinds from Poria cocos, 7 kinds from Rhizoma Atractylodis Macrocephalae, 5 kinds from Rhizoma Zingiberis Recens, and 13 kinds from Radix Paeoniae Alba. There are 2 repeated active components in the compound, therefore, 59 active components were screened out as shown in Table 1.

**Table 1:** Basic information of the active ingredients of Zhenwu Decoction.

Mol ID	Molecule name	OB (%)	DL	Source
MOL002401	Neokadsuranic acid B	43.1	0.85	Monkshood
MOL002434	Carnosifloside I_qt	38.16	0.8	Monkshood
MOL000359	Sitosterol	36.91	0.75	Radix Aconiti Lateralis Preparata and Radix Paeoniae Alba
MOL002397	Karakoline	51.73	0.73	Monkshood
MOL002422	Isotalatizidine	50.82	0.73	Monkshood
MOL002415	6-Demethyldesoline	51.87	0.66	Monkshood
MOL002410	Benzoylnapelline	34.06	0.53	Monkshood
MOL002406	2,7-Dideacetyl-2,7-dibenzoyl-taxayunnanine F	39.43	0.38	Monkshood
MOL002392	Deltoin	46.69	0.37	Monkshood
MOL002398	Karanjin	69.56	0.34	Monkshood
MOL002395	Deoxyandrographolide	56.3	0.31	Monkshood
MOL002388	Delphin_qt	57.76	0.28	Monkshood
MOL000538	Hypaconitine	31.39	0.26	Monkshood
MOL002421	Ignavine	84.08	0.25	Monkshood
MOL002416	Deoxyaconitine	30.96	0.24	Monkshood
MOL002433	(3R,8S,9R,10R,13R,14S,17R)-3-hydroxy-4,4,9,13,14-pentamethyl-17-[(E,2R)-6-methyl-7-[(2R,3R,4S,5S,6R)-3,4, 5-trihydroxy-6-[[(2R,3R,4S,5S,6R)-3,4,5-trihydroxy-6-(hydroxymethyl)oxan-2-yl]oxymethyl]oxan-2-yl]oxyhept-5-en-2-yl]-1, 2,3,7,8,10,12,15,16,17-decahydr	41.52	0.22	Monkshood
MOL002419	(R)-Norcoclaurine	82.54	0.21	Monkshood
MOL002211	11,14-eicosadienoic acid	39.99	0.2	Monkshood
MOL002423	Jesaconitine	33.41	0.19	Monkshood
MOL002393	Demethyldelavaine A	34.52	0.18	Monkshood
MOL002394	Demethyldelavaine B	34.52	0.18	Monkshood
MOL000300	Dehydroeburicoic acid	44.17	0.83	Poria cocos
MOL000285	(2R)-2-[(5R,10S,13R,14R,16R,17R)-16-hydroxy-3-keto-4,4,10,13,14-pentamethyl-1,2,5,6,12,15,16, 17-octahydrocyclopenta[a]phenanthren-17-yl]-5-isopropyl-hex-5-enoic acid	38.26	0.82	Poria cocos
MOL000280	(2R)-2-[(3S,5R,10S,13R,14R,16R,17R)-3,16-dihydroxy-4,4,10,13,14-pentamethyl-2,3,5,6,12,15,16, 17-octahydro-1H-cyclopenta[a]phenanthren-17-yl]-5-isopropyl-hex-5-enoic acid	31.07	0.82	Poria cocos
MOL000283	Ergosterol peroxide	40.36	0.81	Poria cocos
MOL000287	3beta-Hydroxy-24-methylene-8-lanostene-21-oic acid	38.7	0.81	Poria cocos
MOL000276	7,9(11)-dehydropachymic acid	35.11	0.81	Poria cocos
MOL000289	Pachymic acid	33.63	0.81	Poria cocos
MOL000273	(2R)-2-[(3S,5R,10S,13R,14R,16R,17R)-3,16-dihydroxy-4,4,10,13,14-pentamethyl-2,3,5,6,12,15,16, 17-octahydro-1H-cyclopenta[a]phenanthren-17-yl]-6-methylhept-5-enoic acid	30.93	0.81	Poria cocos
MOL000275	Trametenolic acid	38.71	0.8	Poria cocos
MOL000279	Cerevisterol	37.96	0.77	Poria cocos
MOL000290	Poricoic acid A	30.61	0.76	Poria cocos
MOL000292	Poricoic acid C	38.15	0.75	Poria cocos
MOL000296	Hederagenin	36.91	0.75	Poria cocos
MOL000291	Poricoic acid B	30.52	0.75	Poria cocos
MOL000282	Ergosta-7,22E-dien-3beta-ol	43.51	0.72	Poria cocos
MOL000033	(3S,8S,9S,10R,13R,14S,17R)-10,13-dimethyl-17-[(2R,5S)-5-propan-2-yloctan-2-yl]-2,3,4,7,8,9,11,12,14,15,16, 17-dodecahydro-1H-cyclopenta[a]phenanthren-3-ol	36.23	0.78	Atractylodes macroce
MOL000028	α-Amyrin	39.51	0.76	Atractylodes macroce
MOL000021	14-acetyl-12-senecioyl-2E,8E,10E-atractylentriol	60.31	0.31	Atractylodes macroce
MOL000022	14-acetyl-12-senecioyl-2E,8Z,10E-atractylentriol	63.37	0.3	Atractylodes macroce
MOL000020	12-senecioyl-2E,8E,10E-atractylentriol	62.4	0.22	Atractylodes macroce
MOL000049	3β-acetoxyatractylone	54.07	0.22	Atractylodes macroce
MOL000072	8β-ethoxy atractylenolide III	35.95	0.21	Atractylodes macroce
MOL001921	Lactiflorin	49.12	0.8	Radix Paeoniae Alba
MOL001924	Paeoniflorin	53.87	0.79	Radix Paeoniae Alba
MOL000211	Mairin	55.38	0.78	Radix Paeoniae Alba
MOL000358	Beta-sitosterol	36.91	0.75	Radix Paeoniae Alba and Rhizoma Zingiberis Recens
MOL001930	Benzoyl paeoniflorin	31.27	0.75	Radix Paeoniae Alba
MOL001919	(3S,5R,8R,9R,10S,14S)-3,17-dihydroxy-4,4,8,10,14-pentamethyl-2,3,5,6,7,9-hexahydro-1H-cyclopenta[a]phenanthrene-15, 16-dione	43.56	0.53	Radix Paeoniae Alba
MOL001925	paeoniflorin_qt	68.18	0.4	Radix Paeoniae Alba
MOL001910	11alpha,12alpha-epoxy-3beta-23-dihydroxy-30-norolean-20-en-28,12beta-olide	64.77	0.38	Radix Paeoniae Alba
MOL001918	Paeoniflorgenone	87.59	0.37	Radix Paeoniae Alba
MOL001928	Albiflorin_qt	66.64	0.33	Radix Paeoniae Alba
MOL000492	(+)-catechin	54.83	0.24	Radix Paeoniae Alba
MOL000422	Kaempferol	41.88	0.24	Radix Paeoniae Alba
MOL000449	Stigmasterol	43.83	0.76	Ginger
MOL001771	Poriferast-5-en-3beta-ol	36.91	0.75	Ginger
MOL006129	6-methylgingediacetate2	48.73	0.32	Ginger
MOL008698	Dihydrocapsaicin	47.07	0.19	Ginger

DL = drug likeness.

### 3.2. Target prediction of ZWD active ingredient

The 59 core active components of ZWD were input into the TCMSP database to predict 281 protein targets of the active components. By inputting the names of protein targets in UniProtKB function items of Uniprot database, limiting “Reviewed (Swiss-Prot)” and species to “Human,” 229 protein targets were obtained after correcting the names of protein targets and removing duplications. 27 core components and their corresponding 93 protein targets.

### 3.3. Screening of HF-related gene targets

**Figure 1.**
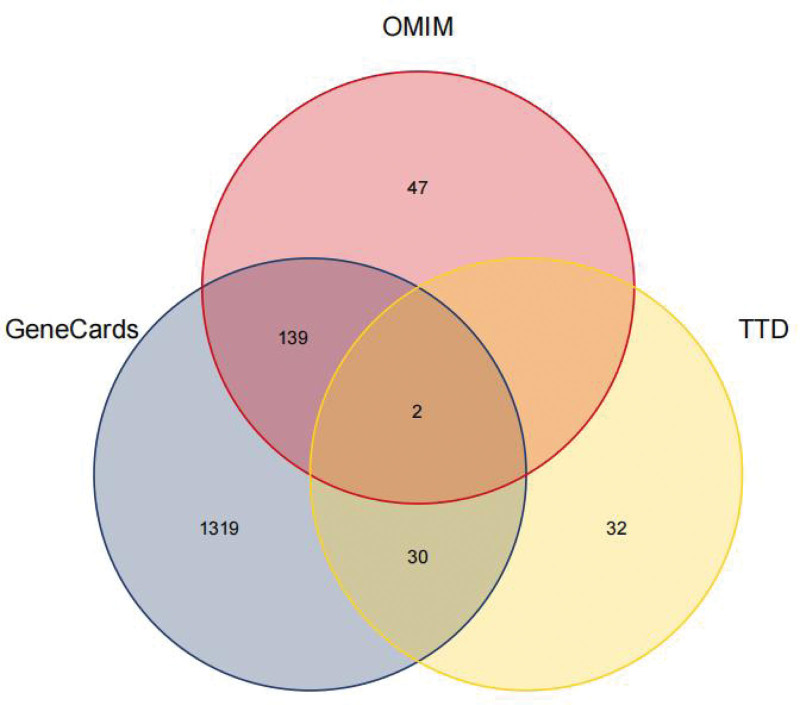
Venn map of disease targets in HF. HF = heart failure.

**Figure 2.**
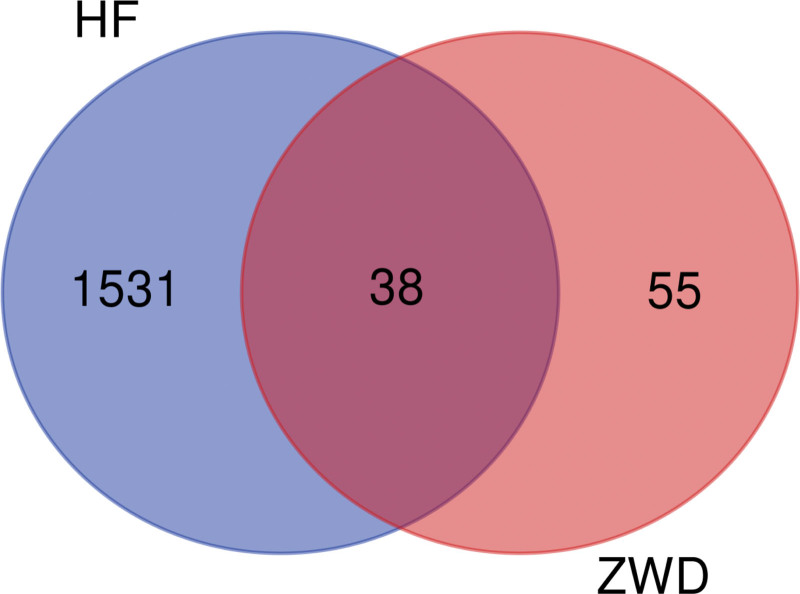
Venn map of the intersection of ZWD targets and HF targets. HF = heart failure.

The Potential targets of HF were predicted in GeneCards, OMIM, TTD and other databases, and the disease targets were mapped by Venny 2.1 website (https://bioinfogp.cnb.csic.es/tools/venny/index.html). 1569 disease targets were obtained by sorting and removing duplicates, as shown in Figure 1; 38 core targets were obtained by mapping the screened disease targets with ZWD active ingredient targets, and Venny plots were drawn, as shown in Figure 2.

### 3.4. Construction and analysis of ZWD active ingredient-HF disease target network

**Figure 3.**
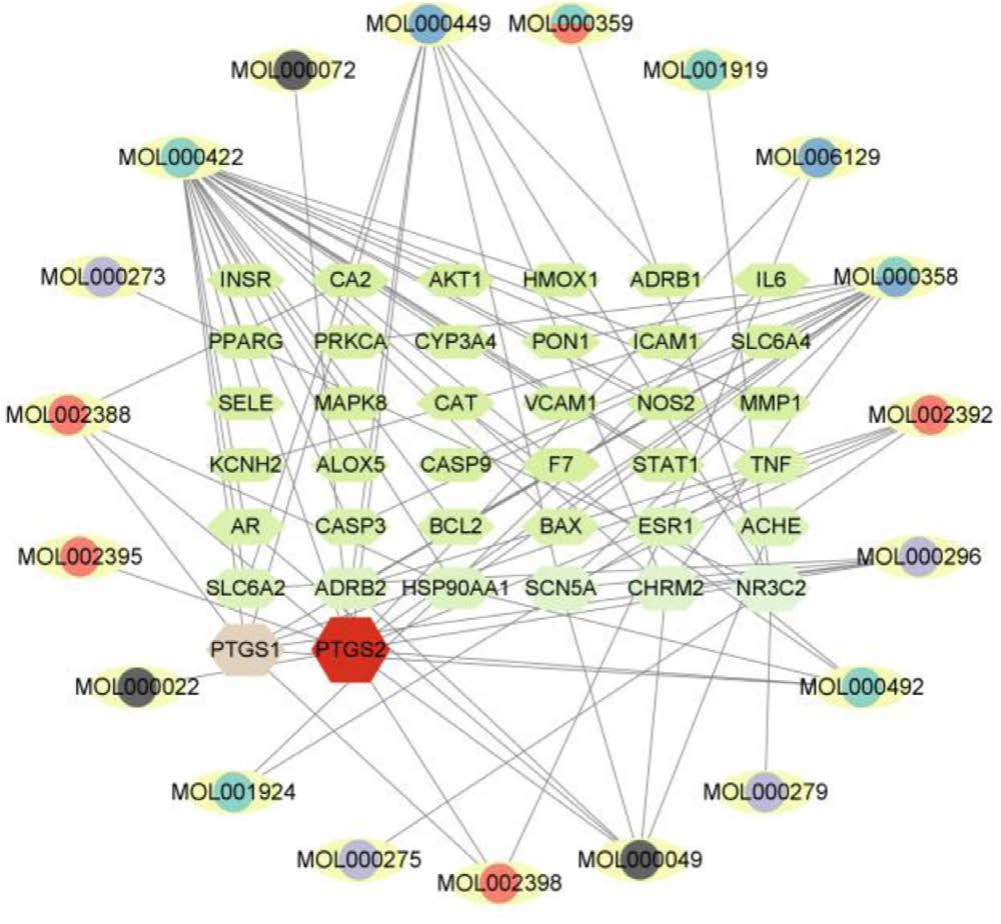
“Drug-active ingredient-disease-target” network diagram.

The 27 active components of ZWD and the 38 predicted core targets were imported into Cytoscape 3.9.1 software to construct a “drug-active component-disease-target” interaction network diagram, as shown in Figure 3, including 57 nodes and 87 edges. The node size in the network graph is proportional to the Degree value. Network Analyzer was used to analyze the network topological parameters of the active ingredients. The results of the network topological parameters of the active ingredients are shown in Table 2. The results show that the Degrees of kaempferol and β-sitosterol are > 10, indicating that the above ingredients are the main active ingredients of ZWD in the treatment of HF.

**Table 2:** Network topology analysis results of main active ingredients (top 10 of degree).

Active ingredient	Degree	Betweenness	Closeness	Source
Kaempferol	25	0.58045	0.509090	Radix Paeoniae Alba
Beta-sitosterol	14	0.22346	0.411764	Radix Paeoniae Alba and Rhizoma Zingiberis Recens
Stigmasterol	8	0.243322	0.405797	Ginger
3β-acetoxyatractylone	6	0.024008	0.368421	Atractylodes macroce
(+)-catechin	5	0.054326	0.363636	Radix Paeoniae Alba
Hederagenin	5	0.010389	0.363636	Poria cocos
Deltoin	5	0.014365	0.363636	Monkshood
Delphin_qt	4	0.038309	0.358974	Monkshood
Karanjin	3	0.012173	0.35443	Monkshood
Paeoniflorin	2	0.035714	0.261682	Radix Paeoniae Alba

### 3.5. Construction and analysis of protein interaction network (PPI)

**Figure 4.**
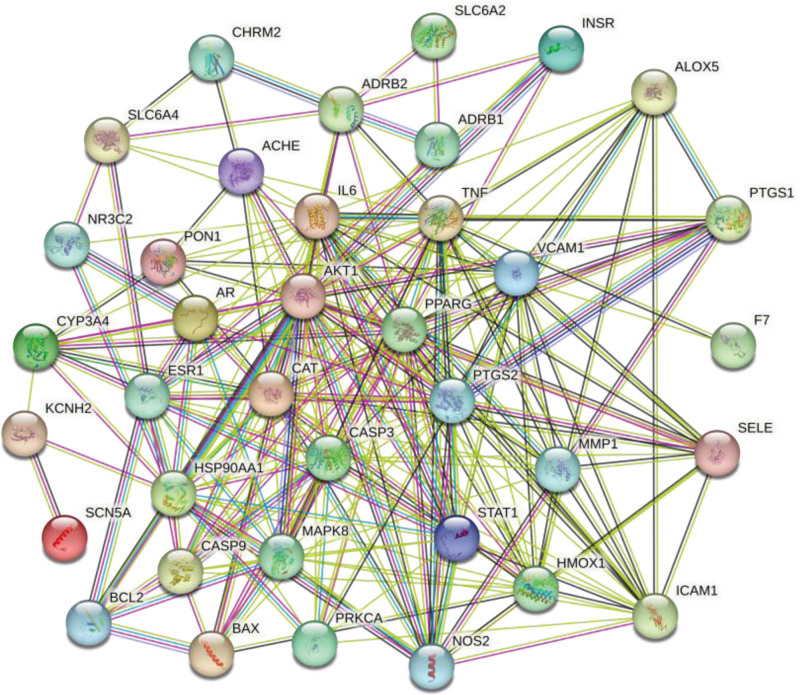
PPI Network Diagram. PPI = protein-protein interaction relationship.

**Figure 5.**
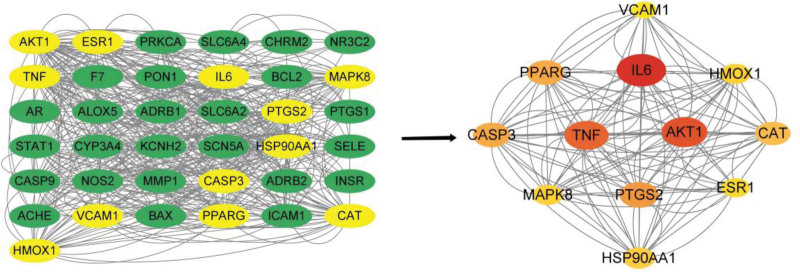
Core target screening process and core target network.

After finding 38 targets at the intersection of traditional Chinese medicine compound ZWD and HF disease and drawing the protein interaction network diagram, the free protein was hidden, and 37 targets were obtained, as shown in Figure 4. Cytoscape 3.9.1 software was used for visualization, in which the size of the node in the network diagram represents the corresponding value, and the larger the node is, the larger the Degree value is, as shown in Table 3. The Cytohubba plug-in was used to analyze the topological parameters of the relevant targets, and a core target protein interaction network containing 12 nodes and 258 edges was obtained, as shown in Figure 5. The results showed that these 12 targets may be potential therapeutic targets for ZWD in the treatment of HF. Degree values of IL-6, AKT1 and TNF were higher than 50, and they were predicted to be the main targets.

**Table 3:** Analysis results of core target network topology (top 15 of degree).

Target	Degree	Betweenness	Closeness
IL6	58	183.6196026	0.818181818
AKT1	54	167.1420912	0.782608696
TNF	52	97.58543679	0.765957447
PTGS2	46	33.74414474	0.705882353
CASP3	44	32.41463536	0.692307692
PPARG	44	64.83871129	0.705882353
CAT	42	33.88615274	0.679245283
HSP90AA1	38	120.8392496	0.654545455
HMOX1	36	9.63969919	0.631578947
MAPK8	36	12.63289488	0.631578947
ESR1	34	41.96863692	0.631578947
ICAM1	32	5.616810967	0.590163934
VCAM1	30	6.745093795	0.580645161
NOS2	30	4.694416694	0.600000000
STAT1	30	4.308752359	0.600000000

TNF = tumor necrosis factor.

### 3.6. GO function and KEGG pathway enrichment analysis

**Figure 6.**
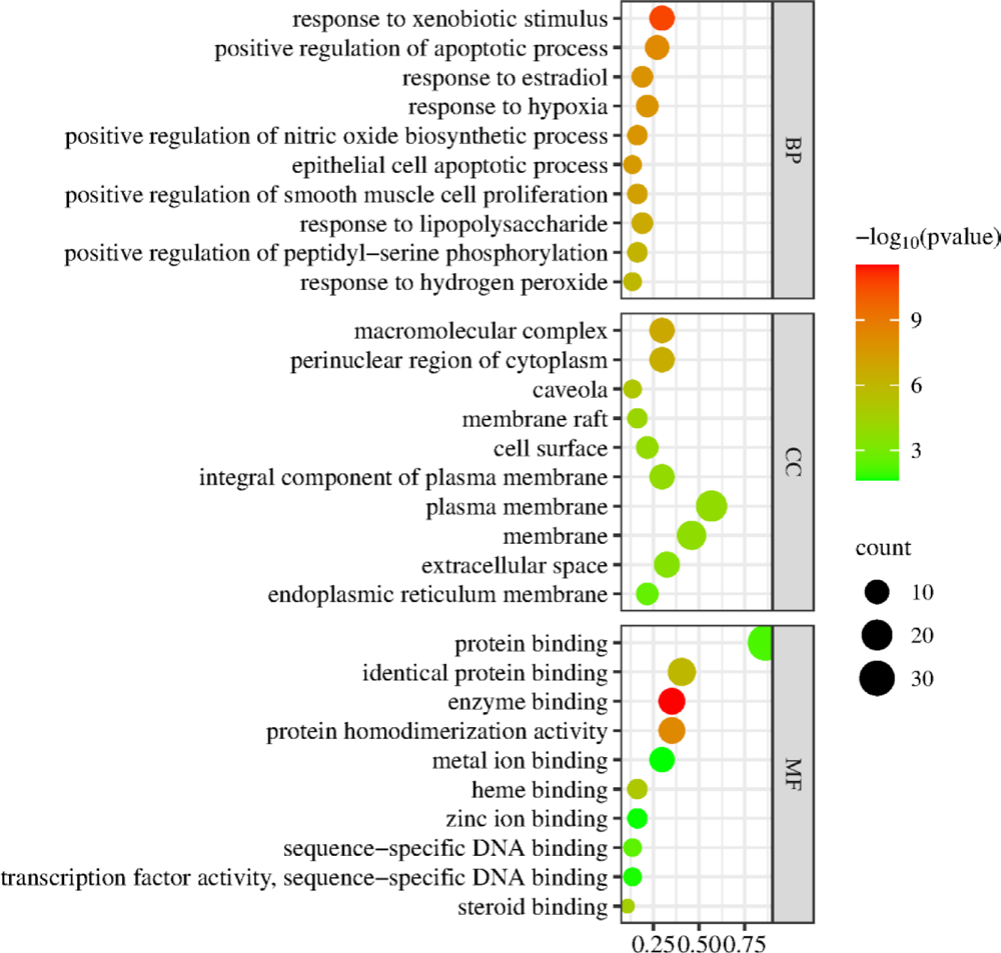
GO enrichment analysis.

The 37 targets of ZWD for HF were imported into the DAVID data platform for GO function and KEGG pathway enrichment analysis. Bubble plots were drawn for the top 10 GO analysis results, including biological function, cellular composition, and molecular function, as shown in Figure 6. The results of go enrichment showed that the active components of ZWD mainly act on the endoplasmic reticulum membrane, extracellular space, cell surface, membrane rafts, etc. Their molecular functions include regulating the response to hydrogen peroxide, the positive regulation of peptidyl-serine phosphorylation, the response to lipopolysaccharide, the positive regulation of smooth muscle cell proliferation, and the response to hypoxia; Biological processes include sequence-specific DNA binding, zinc ion binding, heme binding, enzyme binding, and protein binding.

**Figure 7.**
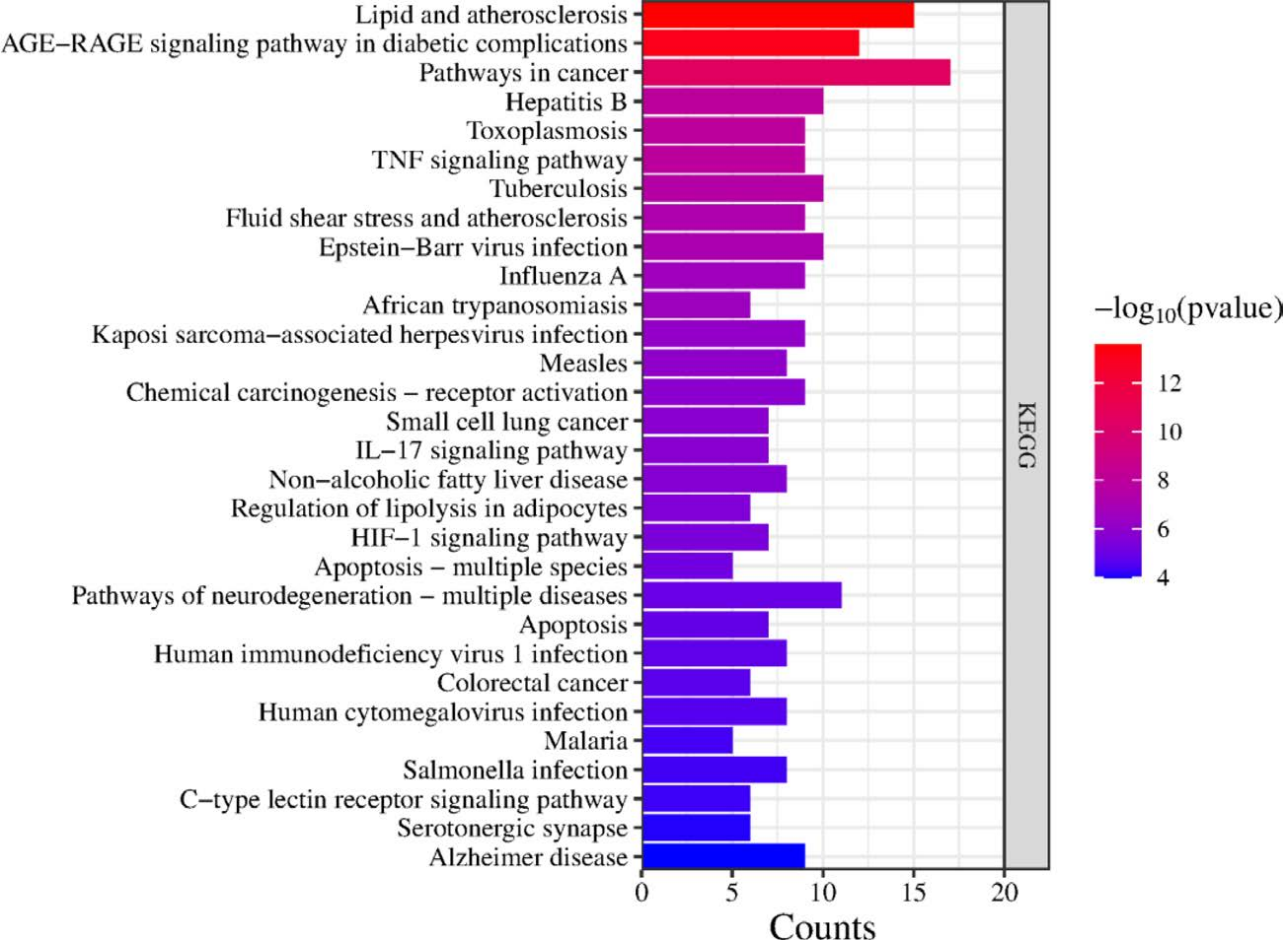
KEGG pathway enrichment analysis. KEGG = Kyoto Encyclopedia of Genes and Genomes.

A total of 110 enrichment messages were obtained by KEGG pathway enrichment analysis. The results showed that the main signaling pathways involved in the targets were lipid and atherosclerosis, AGE − RAGE signaling pathway in diabetic complications, TNF signaling pathway, fluid shear stress and atherosclerosis, IL − 17 signaling pathway and HIF − 1 signaling pathway, etc, to visualize the results of the top 30 enrichment rankings, see Figure 7.

### 3.7. Molecular docking results

The consensus of researchers is that molecular docking binding energy \ binding energy ≤ −5.0 kJ/mol, molecular docking results.^[9]^ The active ingredients with the highest correlation between ZWD active ingredients and HF disease targets were used as molecular docking ligands, and the core gene target proteins obtained from the PPI network were used as molecular docking receptors. AutoDockTools 1.5.7 software was used for molecular docking. The corresponding docking scores are shown in Table 4. The results showed that the docking results between the core active components of ZWD and the core targets were stable.

**Table 4:** Molecular docking results of core targets and core active ingredients for ZWD treatment of HF.

Identifier	Target	Binding energy (/KJ · mol-1)	
		Kaempferol	Beta-sitosterol
4NI7	IL6	−7.5	−7.6
3CQU	AKT1	−7.1	−7.3
3WD5	TNF	−7.4	−7.7

HF = heart failure, TNF = tumor necrosis factor.

## 4. Discussion

Network pharmacology is the study of interactions among drug components, targets, and diseases from the perspective of biomolecular networks. The interaction between the active ingredients of drugs and their targets and the targets of diseases is constructed as a network model, and then the topological parameters of the nodes in the network are evaluated by software to explore the importance of the nodes in the whole network model. Based on the TCMSP database, 59 ZWD active components were obtained with OB > 30% and DL > 0.18 as screening parameters, and 27 active components were obtained after Uniprot database correction and target prediction. The results showed that the activity of Radix Aconiti Lateralis Preparata in ZWD compound was the strongest, followed by Poria cocos, Radix Paeoniae Alba, Rhizoma Atractylodis Macrocephalae and Rhizoma Zingiberis Recens; literature verification was conducted on the screened core components, and it was found that aconitine, one of the main components of Radix Aconiti Lateralis Preparata, was an adrenergic receptor agonist, which could activate adrenergic receptors and thus play a cardiotonic role^[10]^; Kaempferol can inhibit cardiomyocyte apoptosis and oxidative stress of hypoxic cardiomyocytes, and can reduce cellular oxidative stress, inflammation and apoptosis by regulating the 2 signaling pathways of Nuclear Factor E2-related factor 2 and Nuclear Factor-κB (NF-κB) to prevent heart failure^[11,12]^; Both beta-sitosterol and Stigmasterol are phytosterols, which have pharmacological activities such as antioxidant, cholesterol-lowering and anti-inflammatory, and studies have shown that they can reduce the risk of cardiovascular disease^[13–15]^; Experimental studies of paeoniflorin have shown that it can reduce ventricular remodeling and improve cardiac function in rats.^[16]^ Based on the active components and targets retrieved, the potential biological mechanism of ZWD in the treatment of HF was discussed. We obtained 1569 HF-related targets using GeneCards, OMIM and TTD databases, and 38 intersection targets were obtained by mapping with ZWD active component targets. Animal experimental studies have found that Zwd Kezhenwu Decoction can reduce cardiomyocyte apoptosis and myocardial pathological changes in HF rats, enhance myocardial contractility in HF rats, and then delay the process of heart failure in rats. The mechanism may be achieved by inhibiting the expression of AKT1.^[17]^

Through the construction of PPI network, it is found that there is a close and complex relationship between the target proteins. Through the topological analysis of the targets of PPI network, the target proteins of IL6, AKT1 and TNF may be the core targets of ZWD in the treatment of HF. Interleukin (IL-6) and tumor necrosis factor-α (TNF-α) are closely related to the pathogenesis of heart failure. IL-6 and TNF-α can inhibit the contractile function of cardiomyocytes, induce inflammatory activation of macrophages, and stimulate microvascular inflammation and dysfunction. At the same time, IL-6 can promote chronic myocardial fibrosis, which is the pathogenesis of heart failure with preserved ejection fraction.^[18]^ Studies have found that IL6 plays a protective role in the acute response of myocardial cells, but long-term high expression of IL6 can cause decreased myocardial contractility and myocardial hypertrophy.^[19]^ Transient activation of AKT is associated with increased capillary density in the myocardium, and activation of AKT signaling within endothelial cells is sufficient to promote angiogenesis within the myocardium, thereby improving cardiac perfusion.^[20]^ GO functional enrichment analysis of the screened core targets showed that, It involves many biological processes such as endoplasmic reticulum membrane, extracellular space, cell surface, membrane rafts, response to hydrogen peroxide, positive regulation of peptidyl-serine phosphorylation, response to lipopolysaccharide, positive regulation of smooth muscle cell proliferation, response to hypoxia, sequence-specific DNA binding, zinc ion binding, heme binding, enzyme binding and protein binding. Cell composition and molecular function. The main signaling pathways for ZWD treatment of HF include lipid and atherosclerosis, age − RAGE signaling pathway in diabetic complications, TNF signaling pathway, fluid shear stress and atherosclerosis, The interleukin-17 signaling pathway and the hypoxia-inducible factor-1 signaling pathway. Atherosclerosis is the main cause of cardiovascular diseases such as coronary heart disease, heart failure and stroke, and abnormal lipid metabolism, oxidative stress and inflammation are the main characteristics of atherosclerosis^[21]^; Tumor necrosis factor (TNF) is a major mediator of apoptosis, inflammation and humoral immune system, which may be related to the pathogenesis of cardiovascular and cerebrovascular diseases. Many related experiments have shown that the expression level of RAGE in AGE-RAGE cells is usually low. However, when the patient body is in inflammation, trauma, diabetes and other pathological conditions, RAGE may suddenly appear abnormal high level of expression, which will activate NF-κB and other transcription factors, make their expression exceed the normal level, trigger inflammatory toxic reaction, and eventually cause immune tissue dysfunction and structural damage^[22,23]^; The inflammatory response mediated by the il − 17 and HIF − 1 signaling pathways is an important mechanism for the occurrence of cardiovascular events, and its degree of activation is highly related to the HF process; shear stress represents the frictional force exerted by blood flow on the endothelial surface of the vascular wall, plays a central role in vascular biology, and contributes to the development of atherosclerosis.^[24]^ It can be seen that Zhenwu Decoction can achieve the purpose of treating HF by regulating the role of the above pathways.

Molecular docking evaluates the intermolecular interactions by evaluating the binding energy of small molecules and protein ligands in the network, thus improving the accuracy of the network^[25]^. Using molecular docking technology, the binding energy-5.0 kcal/ mol between ligand small molecule and receptor protein, each binding site is formed by stable hydrophobic and hydrogen bonds, where IL 6, AKT 1, TNF and kaempferol and β-sitosterol binding are all relatively stable. The molecular docking results further verified the accuracy of the screened ZWD treatment core network for HF.

## 5. Conclusion

The underlying biological mechanisms of ZWD for HF are characterized by multi-omics, multi-targets, and multi-pathways.IL6, AKT1, and TNF may be the core disease targets of ZWD for HF. It may be related to lipid and atherosclerosis, age − RAGE signaling pathway in diabetic complications, TNF signaling pathway, fluid shear stress and atherosclerosis, interleukin-17 signaling pathway, and hypoxia-inducible factor-1 signaling pathway. In this study, network pharmacology was used to study the mechanism of drug action of ZWD holistically and systematically, which provided a basis for further promotion and application of ZWD. Of course, this study may have some limitations in exploring the active ingredients and targets of ZWD by using only network pharmacology. Therefore, the next step is to carry out experimental research to further confirm the results of this study.

## Author contributions

**Conceptualization:** Sai Yan.

**Data curation:** Sai Yan, Qingchun Shi, Hongtao Ma.

**Formal analysis:** Sai Yan.

**Funding acquisition:** Sai Yan.

**Investigation:** Sai Yan.

**Methodology:** Sai Yan.

**Project administration:** Sai Yan.

**Resources:** Sai Yan.

**Software:** Qian Xu.

**Supervision:** Qian Xu.

**Validation:** Sai Yan.

**Visualization:** Sai Yan.

**Writing – original draft:** Sai Yan.

**Writing – review & editing:** Sai Yan.
